# Introduction of Environmentally Degradable Parameters to Evaluate the Biodegradability of Biodegradable Polymers

**DOI:** 10.1371/journal.pone.0038341

**Published:** 2012-05-31

**Authors:** Wenbin Guo, Jian Tao, Chao Yang, Cunjiang Song, Weitao Geng, Qiang Li, Yuanyuan Wang, Meimei Kong, Shufang Wang

**Affiliations:** 1 Department of Microbiology, Key Laboratory of Molecular Microbiology and Technology for Ministry of Education, College of Life Sciences, NanKai University, Tianjin, China; 2 Key Laboratory of Bioactive Materials for Ministry of Education, College of Life Sciences, NanKai University, Tianjin, China; University of Kansas, United States of America

## Abstract

Environmentally Degradable Parameter (^Ed^ *K*) is of importance in the describing of biodegradability of environmentally biodegradable polymers (BDPs). In this study, a concept ^Ed^ *K* was introduced. A test procedure of using the ISO 14852 method and detecting the evolved carbon dioxide as an analytical parameter was developed, and the calculated ^Ed^ *K* was used as an indicator for the ultimate biodegradability of materials. Starch and polyethylene used as reference materials were defined as the ^Ed^ *K* values of 100 and 0, respectively. Natural soil samples were inoculated into bioreactors, followed by determining the rates of biodegradation of the reference materials and 15 commercial BDPs over a 2-week test period. Finally, a formula was deduced to calculate the value of ^Ed^ *K* for each material. The ^Ed^ *K* values of the tested materials have a positive correlation to their biodegradation rates in the simulated soil environment, and they indicated the relative biodegradation rate of each material among all the tested materials. Therefore, the ^Ed^ *K* was shown to be a reliable indicator for quantitatively evaluating the potential biodegradability of BDPs in the natural environment.

## Introduction

Plastic has many applications in our daily life such as food packaging. Over the last 20 years, the production and consumption of polymeric materials have made plastic pollution a significant environmental issue [1]. It has been estimated that 2% of all plastics eventually reach the environment, thus contributing considerably to a currently acute ecological problem [2]. In addition to causing pollution, the manufacture of plastics consumes oil. As oil resources become increasingly scarce world wide, predictions have estimated that oil reserves are available up to 2040 [3]. Hence, possible oil shortages and plastic pollution have driven the development of biobased and biodegradable polymers (BBDPs) derived from renewable resources [4]. Environmentally biodegradable polymers (BDPs) are kinds of environmentally-friendly materials, which can be degraded into carbon dioxide and water by microorganisms in natural environment. BDPs are sustainable materials with low environmental impacts, low energy consumption and high biodegradability compared to oil-based materials [5].

BDPs can be divided into two classes according to the source they based. One class is non-biobased BDPs such as polyethylene glycol (PEG), polyethylene oxide (PEO) [6], polyester amide (PEA) [7], poly (propylene carbonate) (PPC) [8] and polycaprolactone (PCL) [9]. The other class is biobased BDPs which can be divided into three kinds: microbial polymers such as pullulan, curdlan, polyhydroxyalkanoates (PHA) [10], [11]; chemically synthesized polymers such as poly (butylene succinate) (PBS) [12] and polylactic acid (PLA) [13]; natural polymers such as chitosan, cellulose and polysaccharide [14]–[16].

Just as the petroleum based polymers polyethylene (PE) and polypropylene (PP), BDPs have parameters characterizing their physical and chemical properties, such as melting temperature (*T*_m_), glass transition temperature (*T*_g_), Young's modulus (E), solubility parameter (δ), etc [17]. However, a single parameter describing environmental degradability of BDPs has not yet been defined. Biodegradability has always been considered as an important character for BDPs, but until recently a single environmental degradability parameter (^Ed^ *K*) has not yet been defined.

Methods for measuring biodegradability can be divided into two principal groups: (i) direct measurement of parent BDPs concentrations; (ii) indirect measurement of parent BDPs bioconversion, such as carbon dioxide production [18]–[21]. For practical and legislative purposes, a number of biodegradation test procedures have been standardized to determine the biodegradability of materials. These methods include, among others, ISO 14851, ISO 14852, ISO 14855, ISO 846, ASTM D 5209-91, ASTM D 5247-92, etc. Currently, when a new BDP is synthesized, outdoor and indoor methods are used to evaluate the degradability of the material. These standard methods can perfectly determine whether a certain material is a BDP under certain experimental condition, and give the biodegradation rate of BDPs, but can not manifest their advantages and disadvantages in the aspect of degradability compared with other BDPs. The aim of the present work is to define the environmentally degradable parameter, ^Ed^ *K*, and describe the methods for ^Ed^ *K* determination.

## Materials and Methods

### Ethics Statement

We state that “No specific permits were required for the described field studies.” We state that “No any relevant permissions/permits required for our observational or field studies.” For any locations/activities for which specific permission was not required, we state that a. no specific permissions were required for these locations/activities; b. that the location is not privately-owned or protected in any way and c. that the field studies did not involve endangered or protected species.

### BDPs selected

Fifteen BDPs were selected as the only carbon and energy sources in a mineral salt solution, their names, molecular weights and makers are summarized in Table 1.

**Table 1:** BDPs and reference materials.

Material	*M*_w_	Supplier
Pullulan	*M*_w_ 200,000	Tokyo Kasei Kogyo Co. Ltd., Japan
Curdlan	*M*_w_ 40,000–600,000	Wako Pure Chemical Industries, Ltd., Japan
Chitosan	*M*_w_ 120,000–300,000	Shanghai Boao Co. Ltd., China
Cellulose	*M*_w_ 5,000–250,000	Merck Co. Ltd., Germany
Poly (3-hydroxybutyrate-*co*- 3-hydroxyvalerate), PHBV	*M*_n_ 750,000	*ZENEKA* Co. Ltd., Japan
Poly (3-hydroxybutyrate-*co*- 3-hydroxyhexanoate), PHBHHx	*M*_n_ 160,000	*KANEKA* Co. Ltd., Japan
Poly (ε-caprolactone), PCL	*M*_n_ 50,000	*SHOWA* Co. Ltd., Japan
Poly (butylenes succinate), PBS	*M*_n_ 140,000	*SHYUWA* Polymer Co. Ltd., Japan
Poly (butylenes succinate-*co*- adipate), PBSA	*M*_n_ 140,000	*SHYUWA* Polymer Co. Ltd., Japan
Poly (vinyl alcohol), PVA	*M_n_* 1,750	Shanghai Reagent Corporation, Chinese Medicine Corporation
Poly (ethylene glycol), PEG	*M*_n_ 2,000	Shanghai Reagent Corporation, Chinese Medicine Corporation
Poly (ethylene oxide), PEO	*M*_n_ 100,000	Liansheng Chemical Engineering Ltd. Co., Shanghai, China
Poly (propylene carbonate), PPC	*M*_n_ 200,000	Mengxi High Technol Co. Ltd., China
Poly (lactic acid), PLA	*M*_n_ 200,000	*SHIMADZU* Co. Ltd., Japan
Poly (ester amide), PEA	*M*_n_ 200,000	Chengdu Institute of Organic-chemistry, Chinese Academy of Science
Soluble starch	*M*_w_ 300,000– 3,000,000	Wako Pure Chemical Industries, Ltd., Japan
Polyethylene, PE	*M*_w_ 200,000	Daqing petrochemicals Co. Ltd., Daqing, Heilongjiang Province, China

### Mineral salt solution preparation

ISO14852 method was used to detect the degradability of the above materials, in which two different concentration mineral salt solutions were given. The low one was used to represent the natural environment while the high one could accelerate the microorganism reproduction in the inoculation solution. A mineral salt concentration between the low and high concentrations was used here to simulate the natural environment and to shorten the detection time. The mineral salt solution contained 100 mL/L solution A, 10 ml/L solution B, 1 ml/L solution C and 1 ml/L solutionD and adjusted to pH 7.4. Solution A consisted of (g/L): 8.5 KH_2_PO_4_, 21.75 K_2_HPO_4_, 33.4 Na_2_HPO_4_•12 H_2_O and 0.5 NH_4_Cl. Solution B, C and D contained 22.5 g/L MgSO_4_•7 H_2_O, 36.4 g/L CaCl_2_•2 H_2_O and 0.25 g/L FeCl_3_•6 H_2_O respectively. Preliminary results indicated that this mineral salt solution composition was suitable for the growth of microorganisms and the secretion of degrading enzymes.

### Materials mass determination

Because ISO14852 method required that the total organic carbon (TOC) content of the test material in the bioreactor was in the range of 100–2000 mg/L, and that the C/N mass ratio should be controlled at 40∶1, the TOC was determined to be 520 mg/L according to the TON (13 mg/L) in mineral salt solution. 400 mg starch in 300 mL mineral salt solution yielded a TOC of 518 mg/L, which was conveniently close to the recommendation of 520 mg/L. This TOC was used as the basis of all further tests and weights of the other polymers were calculated accordingly. The total organic carbon content of the test materials determined as the equation below:
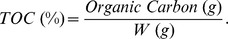



The TOC values of PHBHHx, PHBV, PBSA and PEA were correlated with the monomer content and were provided by the suppliers. The masses for each material added into the bioreactors are shown in Table 2.

**Table 2:** *Xc* of materials and the mass added into bioreactor.

Materials	*Xc* (%) ^a^	m _material_ (mg) ^b^
Starch	38.9	400.0
Pullulan	44.4	350.0
Curdlan	36.3	428.0
PHBHHx	60.0	259.0
PHBV	58.1	268.0
PEA	64.3	242.0
PCL	63.2	246.0
Cellulose	56.3	276.0
Chitosan	40.4	384.0
PEG	54.6	285.0
PVA	54.6	285.0
PEO	54.6	285.0
PPC	47.1	330.0
PBSA	57.8	269.0
PBS	56.8	274.0
PLA	49.5	314.0
PE	85.7	181.0

a: *Xc*: Total organic carbon of each BDPs, expressed in percent. The values of the TOC for each BDP were constant as they were counted from their chemical formulation.

b: m _material_ (mg) : the mass of each BDPs added into the flask, it was calculated by (518 mg/L× 0.3 L)/*Xc.*

### Preparation of inoculation solution

Activated sludge, compost or fertile soil could be used as inoculum mentioned in the standard method. Here, the farmland soil used as inoculum was from Zhouli village, Xiqing district of Tianjin City (collected at a depth of 0–10 cm). The soil used to inoculate the bioreactor was analyzed according to the standard methods [22], and its properties were as follows: pH (H_2_O) 8.20, pH (KCl) 7.90, 17.86% H_2_O, 2.18% C, 2.23% H and 0.85% N. The total number of microorganisms (including bacteria and fungi) in the soil was 6.9×10^7^ per gram of wet soil using direct plate counting method which consisted of evenly spreading the diluted sample over an LB agar plate under aerobic condition at 30°C for 48 hours. LB agar medium contained yeast extract 5 g/L, tryptone 10 g/L, NaCl 10 g/L and agar 15 g/L. Using this method yielded colonies that form on the surface of the agar. The inoculation solution was prepared by adding 100 g soil to 1000 ml sterile mineral salt solution, stirring for 30 min at 4°C. After allowing the solution to stand for 30 min, the upper suspension was made as the inoculum.

### Reference material selection

**Figure 1:**
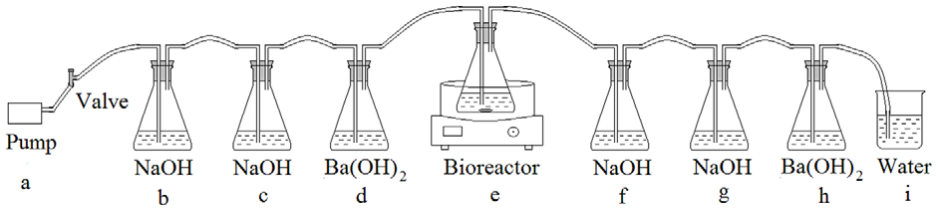
Bioreactor for detection of released CO_2_ by BDPs degradation. a. air pump to provide air flow (50–100 ml/min); b and c. 500 ml flasks filled with 300 ml NaOH solution (10 M), to remove CO_2_ from pumped air; d. 500 ml flask filled with 200 ml Ba(OH)_2_ solution (0.0125 M), to indicate complete removal of CO_2_ in pumped air; e. bioreactor (500 ml flask filled with 300 ml mineral salt solution); f and g. 500 ml flasks filled with 250 ml NaOH solution (0.05 M), to absorb the CO_2_ released in the bioreactor during biodegradation; h. 500 ml flask filled with 200 ml Ba(OH)_2_ solution (0.0125 M), to indicate complete removal of CO_2_ released in the bioreactor; i. water, used to confirm the airtightness of the device. A thermostatic magnetic stirrer was employed to control the temperature and rotation speed in the bioreactor. The material to be tested and inoculation solution were added into the bioreactor flask.

Carbon dioxide from the BDPs was detected and the biodegradation rate was calculated from the ratio of released CO_2_ to the theoretical amount of CO_2_. The apparatus used to detect the release of CO_2_ by BDP degradation is shown in Figure 1. PE and starch were used as reference materials, because PE was not degraded over 32 years [23] and starch could be degraded most easily [24]. We defined the ^Ed^ *K* value of PE as 0, and the ^Ed^ *K* value of starch as 100. Therefore, the ^Ed^ *K* of other BDPs should lie between 0 and 100.

### Determination of testing time required for ^Ed^ *K* determination

To determine the time for ^Ed^ *K* detection, the time for starch complete degradation was measured in preliminary experiments. Results indicated that 400 mg starch could be completely degraded in 14 days in 300 ml mineral salt solution. Therefore, the detection time used for determining the ^Ed^ *K* of other BDPs was fixed at 14 days.

### Degradation Method

The degradation experiments were carried out using the device shown in Figure 1. Atmospheric air (78% N_2_, 21% O_2_ and 0.03% CO_2_) was supplied by an air pump at 50–100 mL/min. Flasks b and c were filled with 300 ml NaOH solution (10 M) to remove CO_2_ from pumped air. Flask d was filled with 200 ml Ba(OH)_2_ solution (0.0125 M) to indicate complete removal of CO_2_ in pumped air. Flask e was the bioreactor filled with 300 ml mineral salt solution. A thermostatic magnetic stirrer was employed to control the temperature and rotation speed in the bioreactor. Flask f and g were filled with 250 ml NaOH solution (0.05 M) to absorb the CO_2_ released in the bioreactor during biodegradation process. Flask h was filled with 200 ml Ba(OH)_2_ solution (0.0125 M) to indicate complete removal of CO_2_ released from the bioreactor. Beaker i was filled with water and used to confirm the airtightness of the devices. Various masses of BDPs calculated to give a TOC of 518 mg/L were added. into the bioreactor containing 300 ml mineral salt solution and 24.0 ml inoculation solution was added also. The mixture reacted for 14 days at 30°C. A blank control and reference materials were also prepared. CO_2_ released was absorbed by NaOH and the consumption of NaOH was determined by titration. All measurements of titration were repeated three times. In the CO_2_ absorption process, NaOH is present in excess and the chemical reaction is:

2NaOH+CO_2_ = Na_2_CO_3_+H_2_O (a).

Then, flasks f and g were pooled together and 10 ml of the NaOH solution was sampled and titrated using 0.05 M HCl. The chemical reaction equations are:

NaOH+HCl = NaCl+H_2_O (b);

Na_2_CO_3_+HCl = NaCl+NaHCO_3_ (c).

Phenolphthalein was used as indicator. Phenolphthalein will turn to colorless from red when the pH of the solution become into neutral. At this point, colligating equation (a), (b) and (c), when CO_2_ is in excess, the reaction is:

NaOH+CO_2_ = NaHCO_3_ (d).

ΔV of NaOH equals 10 ml of NaOH minus the volume of titration HCl used.

## Results

### Introduction of equations

The only carbon source and energy resource in the mineral salt solution were the BDPs being tested. The inoculum was fertile soil suspension. A device capable of detecting CO_2_ release from BDPs during degradation was designed in accordance with ISO14852 (Figure 1). The consumption of NaOH was calculated, from which the amount of released CO_2_ was calculated as in equation (1):
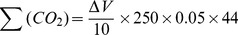
(1)where ∑(CO_2_) is the amount of CO_2_ released by the material, ΔV is volume of NaOH solution consumed (ml), 10 is the sample volume of NaOH (ml), 250 is the total volume of NaOH solution in the absorption bottle (ml) (bottle f and g in Figure 1), 0.05 is the concentration of NaOH solution (M), and 44 g/mol is the molecular weight of CO_2_.

The biodegradation rate or potential biodegradability of BDPs in the natural environment was calculated from the ratio of the amount of CO_2_ released to the maximum theoretical amount of CO_2_ that could be released, as in equation (2):
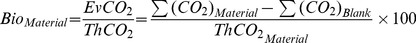
(2)where Bio_Material_ is the biodegradation rate of the test material, Σ(CO_2_)_Material_ is the total amount of CO_2_ released by the material, Σ(CO_2_)_Blank_ is the amount of CO_2_ released in the blank bottle and *ThCO_2_* is the maximum theoretical amount of CO_2_ that could be released.

*ThCO_2_* is calculated as in equation (3):
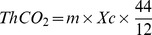
(3)where m is the mass of the material to be determined (g), *Xc* is the organic carbon content of the material to be determined, and 44 g/mol and 12 g/mol are the molecular weight of CO_2_ and atomic weight of carbon respectively. Since TOC was fixed at 518 mg/L and nutrient salt volume was 300 mL for all polymers, *ThCO_2_* was 569.8 mg in all cases.

### Calculation of ^Ed^ *K*

The biodegradation rate of BDPs could be calculated according the following method, the environmentally degradable parameter (^Ed^ *K*) was calculated as in equation (4):
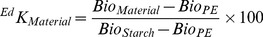
(4)where ^Ed^ *K*_Materials_ is the environmental degradability parameter of the test material, Bio_Starch_ is the biodegradation rate of starch, Bio_Material_ is the biodegradation rate of the test material, and Bio_PE_ is the biodegradation rate of PE.

Combining equations (2) and (4), the environmental degradability parameter is calculated as in equation (5):



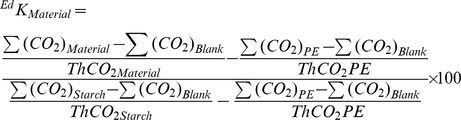
(5)


Because the TOC content of PE, starch and the test materials were identical, so their theoretical amounts of CO_2_ were the same. Meanwhile, the amount of CO_2_ released by PE was equal to that of the blank control where the only carbon source was derived from the inoculation solution. Hence, PE was not degraded at all in the inoculated mineral salt solution. Therefore, equation (5) can be simplified as in equation (6):



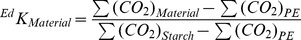
(6)


Combining equations (1) and (6) yields equation (7), the *^Ed^K* value of test materials can be calculated by measuring the consumption of NaOH solution by CO_2_ released during degradation of the material:
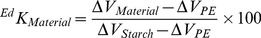
(7)where *Δ* V_Material_ is the volume of NaOH solution consumed by CO_2_ released during the degradation of test material, *Δ*V_Starch_ is the volume of NaOH solution consumed by CO_2_ released during the degradation of starch, and *Δ*V_PE_ is the volume of NaOH solution consumed by CO_2_ released during the degradation of PE.

Fifteen different BDPs were detected by this method, and *ThCO_2_*, CO_2_ released, biodegradation rate and ^Ed^ *K* were listed in Table 3.

**Table 3:** Biodegradation rate and ^Ed^ *K* of BDPs.

Materials	ThCO2 (mg)^a^	EvCO2 (mg)^b^	Biodegradation rate (%)^c^	EdK ^d^
starch	569.8	446.40±9.92	78.34±1.74	100.00±0.00
pullulan	569.8	420.37±7.70	73.77±1.35	94.22±3.74
curdlan	569.8	368.12±10.19	64.60±1.79	82.50±3.13
PHBHHx	569.8	354.20±5.04	62.16±0.88	79.36±1.28
PHBV	569.8	304.70±3.81	53.47±0.67	68.29±2.28
PEA	569.8	206.80±10.49	36.29±1.84	46.34±2.58
PCL	569.8	146.30±3.97	25.68±0.7	32.77±0.21
cellulose	569.8	142.45±3.43	25.00±0.6	31.93±1.36
chitosan	569.8	84.70±2.40	14.86±0.42	18.98±0.71
PEG	569.8	68.20±2.20	11.97±0.39	15.28±0.42
PVA	569.8	28.60±2.52	5.02±0.44	6.41±0.53
PEO	569.8	21.45±1.46	3.76±0.26	4.81±0.43
PPC	569.8	19.25±1.46	3.38±0.26	4.31±0.27
PBSA	569.8	16.50±1.46	2.90±0.26	3.70±0.32
PBS	569.8	11.00±0.55	1.93±0.10	2.47±0.16
PLA	569.8	5.50±1.46	0.97±0.26	1.23±0.32
PE	569.8	0.00±0.00	0.00±0.00	0.00±0.00

a: *ThCO_2_*: the theoretically released CO_2_ of each material; b: *EvCO_2_*: The average with standard deviation of three independent titration results for the actually released CO_2_ of material to be determined in the degradation process; c: Biodegradation rate: the value of biodegradation rate calculated via formula 2; d:^ Ed^ *K*: the degradable parameter calculated via formula 7.

## Discussion

In our, pre-exprement we observed that the amount of CO_2_ released by PE was equal to that of the blank control where the only carbon source was derived from the inoculation solution. PE was not degraded at all in the inoculated mineral salt solution. From the calculation of ^Ed^ *K*, we can see that the values of blank control will be removed as it is a common factor. From equation (7), we only need to know the ΔV of material, PE and starch to calculate ^Ed^ *K*.

Among 15 BDPs tested, the ranking of some BDPs according to the ^Ed^ *K* values was consistent with some of the conclusions in the aspect of biodegradability reported in the literatures although the methods used were different. Rosa *et* *al*. reported that PHBV was the most biodegradable and PCL the least, when PHB, PHBV and PCL were buried in soil compost at pH 11.0 [2]. In our study, the ^Ed^ *K* values of PHBV and PCL were 68.29±2.28 and 32.77±0.21 respectively (Table 3). It indicated that the biodegradability of PHBV used in the study was better than that of PCL, which was in accordance with Rosa *et* *al*.'s conclusion although PHBV used may be different in monomer composition. It was interesting that the soil sample used in this study and Rosa et al.'s study were different. Therefore, investigation of the relationship between the microbial composition and the degradation of BDPs needs to be done in the future studies. According to Song *et* *al.*'s study, PLA belonged to the slow biodegradation rate plastic with mass loss<5% after 90 days [25]. This was also in accordance our observation that PLA had a biodegradation rate of 0.97±0.26 % with the ^Ed^ *K* value of 1.23±0.32 (Table 3).

Fifteen BDPs were detected by this method, and *ThCO_2_*, CO_2_ released, biodegradation rate and ^Ed^ *K* were listed in Table 3. As expected, ^Ed^ *K* of natural polymers was higher than synthetic polymers. Higher ^Ed^ *K* values indicate faster biodegradation rate of the material in the natural environment. The ^Ed^ *K* of PLA was only 1.2, which was consistent with our previous studies that indicated that PLA was not significantly degraded when buried in soil for 3 years [26]. As indicated in ISO14852, employing microorganisms from different sources yields different degradation results. If the detection system could be unified by using a defined inoculum, for example a mixed solution of pure microorganism with prescribed concentrations, just as Guo *et* *al*. [27] described, then the same ^Ed^ *K* of BDPs could be obtained in any laboratory of the whole world.

In this study, a concept of Environmentally Degradable Parameter (^Ed^ *K*) was introduced. A formula was deduced to calculate the value of ^Ed^ *K* for 15 commercial BDPs. The ^Ed^ *K* was shown to be a reliable indicator for quantitatively evaluating the potential biodegradability of BDPs in the nature environment.
